# Effect of Matrix and Graphite Filler on Thermal Conductivity of Industrially Feasible Injection Molded Thermoplastic Composites

**DOI:** 10.3390/polym11010087

**Published:** 2019-01-08

**Authors:** Tom Wieme, Lingyan Duan, Nicolas Mys, Ludwig Cardon, Dagmar R. D’hooge

**Affiliations:** 1 Centre for Polymer and Material Technologies, Department of Materials, Textiles and Chemical Engineering, Ghent University, Technologiepark 915, B-9052 Zwijnaarde (Ghent), Belgium; tom.wieme@ugent.be (T.W.); lingyan.duan@ugent.be (L.D.); Nicolas.Mys@ugent.be (N.M.); 2 Laboratory for Chemical Technology, Department of Materials, Textiles and Chemical Engineering, Ghent University, Technologiepark 914, B-9052 Zwijnaarde (Ghent), Belgium; 3 Centre for Textile Science and Engineering, Department of Materials, Textiles and Chemical Engineering, Ghent University, Technologiepark 907, B-9052 Zwijnaarde (Ghent), Belgium

**Keywords:** thermal conductivity, skin-core layer, injection molding, crystallinity

## Abstract

To understand how the thermal conductivity (TC) of virgin commercial polymers and their composites with low graphite filler amounts can be improved, the effect of material choice, annealing and moisture content is investigated, all with feasible industrial applicability in mind focusing on injection molding. Comparison of commercial HDPE, PP, PLA, ABS, PS, and PA6 based composites under conditions minimizing the effect of the skin-core layer (measurement at half the sample thickness) allows to deduce that at 20 m% of filler, both the (overall) in- and through-plane TC can be significantly improved. The most promising results are for HDPE and PA6 (through/in-plane TC near 0.7/4.3 W·m^−1^K^−1^ for HDPE and 0.47/4.3 W·m^−1^K^−1^ for PA6 or an increase of 50/825% and 45/1200% respectively, compared to the virgin polymer). Testing with annealed and nucleated PA6 and PLA samples shows that further increasing the crystallinity has a limited effect. A variation of the average molar mass and moisture content is also almost without impact. Intriguingly, the variation of the measuring depth allows to control the relative importance of the TC of the core and skin layer. An increased measurement depth, hence, a higher core-to-skin ratio measurement specifically indicates a clear increase in the through-plane TC (e.g., factor 2). Therefore, for basic shapes, the removal of the skin layer is recommendable to increase the TC.

## 1. Introduction

Thermally conductive polymers are of great interest for a vast amount of applications, including heat sinks for light-emitting diodes, batteries and other electronic devices. They are also promising for heat exchange processes that can profit from reduced weight and improved corrosion resistance compared to conventional metal-based heat exchangers [1]. Significant lab-scale research has already been conducted on the improvement of the thermal conductivity (TC) of polymer composites. Focus has been on the use of fillers such as carbon fibers [2,3], graphite and carbon nano particles [2,4,5,6], metals [7,8,9], and more recently carbon nanotubes [10,11] and grapheme [12,13] for applications that allow electrical conductivity and fillers such as boron nitride, aluminum nitride [14], ceramics [15], and metal oxides [16] for non-electrical conductive applications. Typical conductivity values are between 0.15 and 0.50 W·m^−1^K^−1^, which are lower than the conventional metal counterparts with values around 10–5 × 10^2^ W·m^−1^K^−1^ [1]. A high filler content, often more than 35% in volume [17,18], is required in order to improve the TC of lab-scale polymer composites. Only at high filler amounts, a continuous thermal conductive path of fillers can be formed. This critical loading level is called the thermal percolation threshold to differentiate e.g., from an electric percolation threshold [19]. Fillers with a high aspect ratio require lower loading levels to reach this threshold, and carbon nanotubes can help to form conductive connections between the fillers [20,21].

The TC of a composite is in general determined by the TC of the matrix and strongly influenced by the number of interfaces, making, for a given amount, fillers with a high surface area more favorable. It is postulated that polymers with a higher crystallinity possess a better TC, though there are plenty of exceptions as other factors such as side groups, (average) molar mass, bond strength, and processing conditions also play a role [1,10,17,22]. The relevance of processing conditions in view of TC design is less studied but essential, as it can contribute to anisotropy and result in brittle materials with reduced mechanical properties [17]. The most commonly used processing methods for thermoplastic polymers, i.e., injection and extrusion, are known to cause the fillers to orientate with the main flow direction. This results in a high in-plane TC and a low through-plane TC [23,24].

Although the effect of crystallinity has been studied theoretically [25] and at a lab scale [26], almost no data is available on the effect of crystallinity on the TC on an industrial applicable scale. The mismatch in TC during processing can also be expected to be enhanced under industrial scale conditions, which aim at high throughputs, with the same being true for other polymer composite characteristics. In particular, stronger morphological changes are likely. For example, in injection molding, the skin-core effect causes fillers close to the mold wall to show strong orientation with the flow direction, while the fillers in the core are more randomly orientated [27,28,29], though a specific core pattern has been claimed as well [30]. As manipulation or control over orientation can strongly influence the in-plane and through-plane TC [31], further investigation of the skin-core effect seems appropriate. Furthermore, to the best of the authors’ knowledge, a very limited number of studies with both in-plane and through-plane TC measurements have been devoted on a scale transcending the lab scale [23,24,32]. These lab scale studies typically focus on the effect of aspect ratio [33], the combination of different fillers [34] and a comparison with theoretical models [18] rather than the explicit evaluation of the effect of the skin-core formation and matrix related variations on the TC.

In this work, the effect of average molar mass, crystallinity and filler size is therefore studied at a (semi)-industrial manufacturing scale, considering commercial polymers. Since a manifold of thermal management applications are subjected to moisture, the effect of moisture on TC is investigated as well. The focus is on samples prepared with injection molding, as it is one of the most common methods and more scalable techniques for polymer processing, and graphite fillers to obtain a simple and relatively inexpensive, thus industrially relevant manufacturing procedure. Specific focus is on the identification of the most optimal polymer matrix and the effect of skin and core layers on the TC. It is shown that these layers have a significant effect on the TC and the effective reduction of the thickness or modification of the filler spatial arrangement can strongly improve the through-plane TC, at least for basic shapes.

## 2. Materials and Methods

### 2.1. Materials

Flake graphite (Asbury, 3806) with an average size of 19 micron was compounded with different matrices: polypropylene (PP; Sabic, 575P), high density polyethylene (HDPE, Dow, 25055E), acrylonitrile-butadiene-styrene (ABS, LG, HI-121), polyamide-6 (PA6; Solvay, Technyl C 230 Natural), polystyrene (PS; Ineos, Styrolution 165N and 124N), and poly(lactic acide) (PLA; Natureworks, 3100HP). Nucleating agent (Bruggolen P252), kindly supplied by Bruggemann Chemical, Heilbronn, Germany, was used to investigate the effect of crystallinity.

### 2.2. Manufacturing Approach

**Figure 1:**
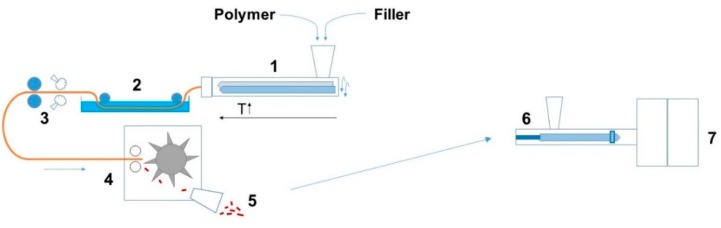
Semi-industrial scale processing of thermally conductive composites includes compounding (**left**) and injection molding (**right**) with (1) twin screw extruder; (2) water bath; (3) drying and pulling; (4) pelletizer; (5) pellets; (6) injection molding machine; and (7) mold.

The overall manufacturing procedure is highlighted in Figure 1. For each polymer matrix, compounds with 0, 10 and 20 m% of the flake graphite filler were produced. Additionally, PLA and PA6 filaments with 0.2, 0.5 and 1.0 m% nucleating agent (N.A.) were produced without fillers. Prior to this, the PLA was dried overnight at 60 °C, while PA6, ABS and PS were dried at 80 °C. The carbon fillers were compounded with thermoplastics using a co-rotating twin screw extruder (APV MP19TC-40 Baker, Peterborough, UK) with variable temperature profiles for different matrices (e.g., for PA6 from 220 °C at the inlet to 250 °C at the die). The extruded filaments were directly cooled in a water bath at room temperature and chopped in pellets. Prior to injection molding, the pellets were dried overnight. The composites were molded into tensile bars according to ISO 527-2 specimen type A, using an injection molding machine (Engel e-victory, Schwertberg, Austria). The dosing speed was maintained as low as possible to avoid damage to the graphite filler.

**Figure 2:**
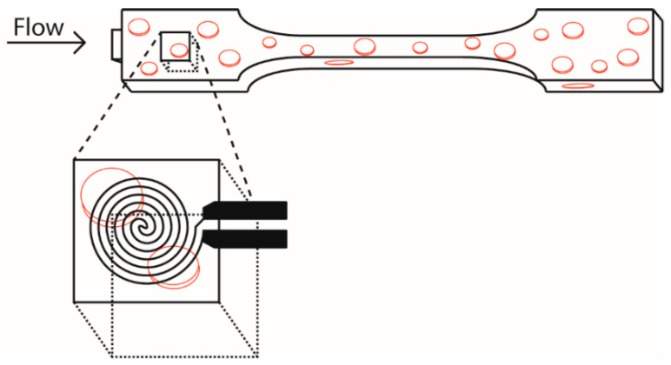
Thermal conductivity (TC) measurement is performed on the injection molded part of the dogbone closest to the gate; cf. Figure 1 (right).

Prior to characterization, all samples were conditioned in a climate controlled room at 23 °C and relative humidity of 50% for at least 48 h. In order to measure the TC, the wide parts of the dogbones were cut off and a cube with a flank size of 10 mm was cut out of the middle of the dogbone, as shown in Figure 2 (closest to the gate). The samples were grinded on sandpaper (grit 300) on one side till the surface was smooth to assure good contact between sensor and sample.

In order to further investigate the effect of crystallinity, PLA samples were tested as prepared and after annealing (–a-annotation in sample notation). The annealing treatment consisted of treating the samples, after injection molding, for 2 h in a furnace at 80 °C and slowly letting them cool down to room temperature. Before testing the TC, these samples were sanded and conditioned as all previous samples. PA6 samples were annealed for 5 h at 175 °C and slowly cooled to room temperature. The effect of moisture on TC was performed by confronting PA6, known as a hygroscopic material, under different humidity conditions before measuring. PA6-samples were installed in a drier at 80 °C for 24 h. Afterwards, they were put in moisture resistant bags and cooled down to 23 °C before measuring. Other samples were placed for a week in distilled water at 23 °C before measurement. The reference samples were measured under regular conditions as mentioned before (23 °C and 50% relative humidity).

### 2.3. Characterization

**Figure 3:**
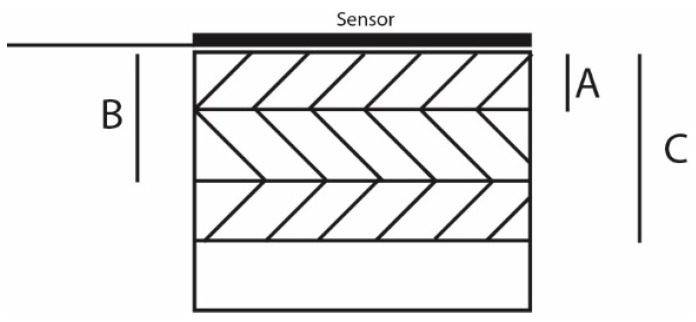
Different measurement times result in the average TC over increasing volumes. Short measurement times result in low probing depth (A) and will thus give an average TC over that measured volume. Longer measurement times (B and C) will give an average TC over larger volumes.

The density of the samples was measured by buoyancy in air and ethanol, using a Precisa XR 205SM-DR balance (Precisa, Dietikon, Switzerland). Heat capacity was measured using the Hot Disk TPS 2500S (Hot Disk, Göteborg, Sweden) heat capacity module with the gold cell reference. All TC numbers are an average of three measurements with acceptable standard deviations (below 5%), unless mentioned otherwise. TC in the in- and through-plane direction were measured using the anisotropic module according to the ISO 22007-2 norm. To compensate for the skin-core effect, the probing depth for the measurements aiming at overall TC values (most measurements) was as close as possible to half the sample thickness (*B* = 2 mm in Figure 3). Isotropic bulk samples were measured using the isotropic module.

The probing depth can be varied by adjusting measurement times so that the skin-layer effect can be explicitly studied alongside the determination of overall TC values. Longer measurements allow the heat wave to travel further, thus allowing to measure TC over a larger volume, as can be seen in Figure 3. Measuring for a short time results in a low measuring depth (A), and will measure the TC of what is mostly the skin layer. At half of the sample thickness (B), the overall value results assume that the morphology is identical for both sides of the sample. Longer measurements (C) give values of the TC of a larger sample volume and will relatively contain more core layers.

The crystallinity of the semi-crystalline polymers (*x*_c_) was measured using differential scanning calometery (DSC; Netzsch Polyma, Selb, Germany). Different maximum temperatures were chosen for different polymers, but heating rate and starting temperature were maintained constant at 10 °C min^−1^ and 30 °C. Peak surface and temperatures were determined using the Protheus software. Data from the first heating run was used. Crystallinity was calculated using [35,36]:

$$x_{c}\;\left(\%\right)=\frac{\Delta H_{\mathrm{melt}}-\Delta H_{\mathrm{cc}}}{\Delta H_{m}^{0}}100$$

in which $\Delta H_{m}$ stands for melt enthalpy, $\Delta H_{\mathrm{cc}}$ for cold crystallization or post-crystallization enthalpy, and $\Delta H_{m}^{0}$ for the theoretical melt enthalpy of 100% crystalline material. The values for $\Delta H_{m}^{0}$ are displayed in Table 1.

Scanning electron microscopy (SEM) images were taken using a Phenom desktop SEM gen 1 (Phenom-world, Eindhoven, The Netherlands). No gold sputtering was required since a sample holder with charge reduction (low vacuum) was used. Samples were frozen in liquid nitrogen before being fractured.

## 3. Results and Discussion

In this section, the effect of process variables such as average molar mass, crystallinity, the presence of moisture, and filler size/amount is investigated, considering the semi-industrial scale manufacturing approach in Figure 1 and focusing both on overall TC values and TC values validating skin-core layer formation. It is reminded that all TC values are averages obtained after statistical analysis.

### 3.1. Effect of Average Molar Mass

One could argue that a higher average molar mass should improve the TC of a polymer matrix, since the TC along chains is higher than that between chains. To test this theory, the TC of two commercial grades of polystyrene (Ineos styrolution 124N (lower average molar mass) and Ineos styrolution 165N (higher average molar mass) were measured. These grades of polystyrene were chosen as they are amorphous, meaning that crystallization effects can be eliminated.

Table 2 shows the recorded (overall) TC values. Focus is on isotropic measurements, as no fillers are present. There seems to be no clear difference in the TC of both polymers but this could be within the error range of the measurement equipment. Hence, based on the data in the present work, it seems that the average molar mass plays at most a very minor role in the TC. This is likely because, besides molar mass, the orientation of the chains and chain entanglement could counter this effect, or perhaps the difference in molar mass for the selected commercial polystyrenes is insufficient.

### 3.2. Effect of Crystallinity

Table 3 shows the relation between TC and crystallinity for several commercial polymers with no nucleation agent (N.A.) added. The amorphous polymers clearly show a lower TC than the semi-crystalline polymers. However, the effect of crystallinity should not be overestimated. While PP has a crystallinity of 44%, the TC is well below that of PA6, with only a crystalline fraction of 16%. This could be caused by the relatively low crystal density and the methyl groups on the polypropylene causing phonon scattering [17]. Other factors such as crystal size, polymer backbone composition and molar mass could also influence the TC of the virgin polymers.

In order to further investigate the effect of crystallinity, samples of PLA and PA6 were produced with N.A. and measured before and after annealing treatments. The N.A. works well for both PA6 and PLA, as can be seen in Table 4. An increase in crystallinity for both PA6 as PLA is visible upon its introduction, already at a mass concentrations as low as 0.2 m%. Higher amounts of N.A. show little additional improvement. Annealing treatments further improve the crystallinity of both PLA and PA6. For PA6, a new DSC peak around 195 °C appears after annealing. This peak can be ascribed to the γ crystalline state of PA6 [39], while the peak around 224 °C can be ascribed to the α crystals. The DSC run of PLA shows no cold crystallization after the annealing treatment (see spectra in the Supporting Information). For PLA with N.A., however, only a (very) small increase in TC can be noticed with increasing crystallinity. It should be mentioned though that this small increase in TC will barely have an effect on the cooling time within the injection process or result in an improved efficiency for heat exchange applications. The crystallinity of such products should thus be tailored for mechanical properties rather than TC. A small decrease in TC can be noticed for PA6, even though crystallinity increases with added N.A. This could possibly be explained by the increased amount of crystals, hence, reduced size of the crystals increasing the number of interfaces between amorphous and crystalline phase or two crystal phases, which has a negative impact on the TC. Annealed PA6 shows slightly increased TC compared with their non-annealed counterpart, since annealing allows crystals to grow in size, reducing the amount of the aforementioned interfaces.

### 3.3. Effect of Moisture

After injection molding and sanding, the PA6 samples were conditioned at 23 °C with 50% relative humidity, while the other samples were maintained in distilled water at 23 °C, hence, wet, and some samples were dried at 80 °C; all for at least 24 h. Before measuring the dried samples, they were cooled down to 23 °C in moisture-tight plastic bags. The results are given in Table 5. There seems to be only a very little difference in TC between the different conditions. This difference will not have severe implications on the performance of thermal management systems, which is a positive finding.

### 3.4. Effect of Filler Amount and Matrix Type

The in-plane and through-plane (top/bottom part) TCs for several virgin polymers and their composites are shown in Table 6, with filler amounts up to 20 m%. These results, which include absolute data and relative data with respect to the virgin polymer, clearly show that the in-plane TC increases more rapidly than the through-plane TC with increasing filler content. This phenomenon can be explained by the manufacturing method. Injection molding is a process that causes the polymer chains to flow, therefore causing the flake fillers to orientate with the flow. The heat flow in the in-plane direction has to cross less filler-matrix interfaces, which is beneficial for TC. Moreover, the TC of graphite as such is anisotropic, with a higher “in-plane” than “through-plane” TC.

There seems to be no clear connection between the in-plane composite TC and the TC of the matrix (top part of Table 6). This is likely because the flow behavior and the related processing parameters, which are different for each polymer, will determine how well the graphite platelets are aligned and therefore how high the TC shall be. Compatibility seems to also play little role in TC of the composites for in-plane measurements. For example, PE shows low compatibility with most fillers [40], while HDPE composites show amongst the highest TCs both in the in- and through-plane direction in Table 6. A simple conclusion for through-plane TC (bottom part of Table 6) is also difficult to make. Most promising are HDPE and PA6 with a through-plane TC near 0.5 W·m^−1^K^−1^ and higher at 20 m% filler. This might be partially due to the already relative high TC of the virgin matrix, as PS shows a similar relative increase but has a lower initial TC. On the other hand, the increase in through-plane TC of ABS and PS is about relatively the same. In some cases (e.g., PP), a decrease in through-plane TC can be noticed with an increasing amount of filler as well. This could be explained by the increasing number of interfaces, causing more phonon scattering and thus reduced TC. The relative low TC of the through-plane direction of graphite, the short distance, and the high number of interfaces are dominant factors under such conditions, causing the TC to drop instead of increase.

Most promising for small electronics heat sinks are modified HDPE, PA6 and PLA, with large in-plane TCs and acceptable through plane TCs, if process design is done properly. Compared to other state of the art options, the modified HDPE outperforms a PP-carbon fiber-carbon nanotube composite [41] in both in-plane and through-plane TC with less filler at a likely lower production price. Through-plane TC of PA6 with 20 m% of filler approaches the through-plane TC of the previously mentioned composite, while heavily outperforming it in in-plane TC. As the previously mentioned PP-based composite was suitable for cooling LEDs, composites presented in this work will likely perform likewise. For heat exchanger applications, performance would strongly depend on thickness of the used components.

### 3.5. Relevance of Morphology: Skin-Core Effect

As seen above (Table 2, Table 3, Table 4, Table 5 and Table 6), probing depth was regulated (ca. half the sample thickness) to minimize the impact of the skin-core effect and thus obtain an overall in/through-plane TC. However, an important research task is the identification of the TC gradient along the thickness, which is covered in the present subsection. By adjusting the measurement time on the Hot Disk TPS 2500 s, the average TC over different depths can be measured. The TCs at different probing depths for PLA and ABS with 20 m% of graphite filler (optimal amount from Table 6) are given in Table 7. The influence of the skin-core effect on the TC of composites becomes very clear here. Upon measuring at a small probing depth, the skin layer is mainly measured. This layer is known for its strong orientation with the flow, where TC in the through-plane will be at a minimum and in-plane TC will be higher. As the probing depth increases, the fraction of the core layer compared to the skin layer will increase, displaying an increased through-plane TC traded off for a decrease in in-plane TC. By increasing the probing depth of ABS, a through-plane TC value twice as high as the value at half the thickness of the sample is found.

**Figure 4:**
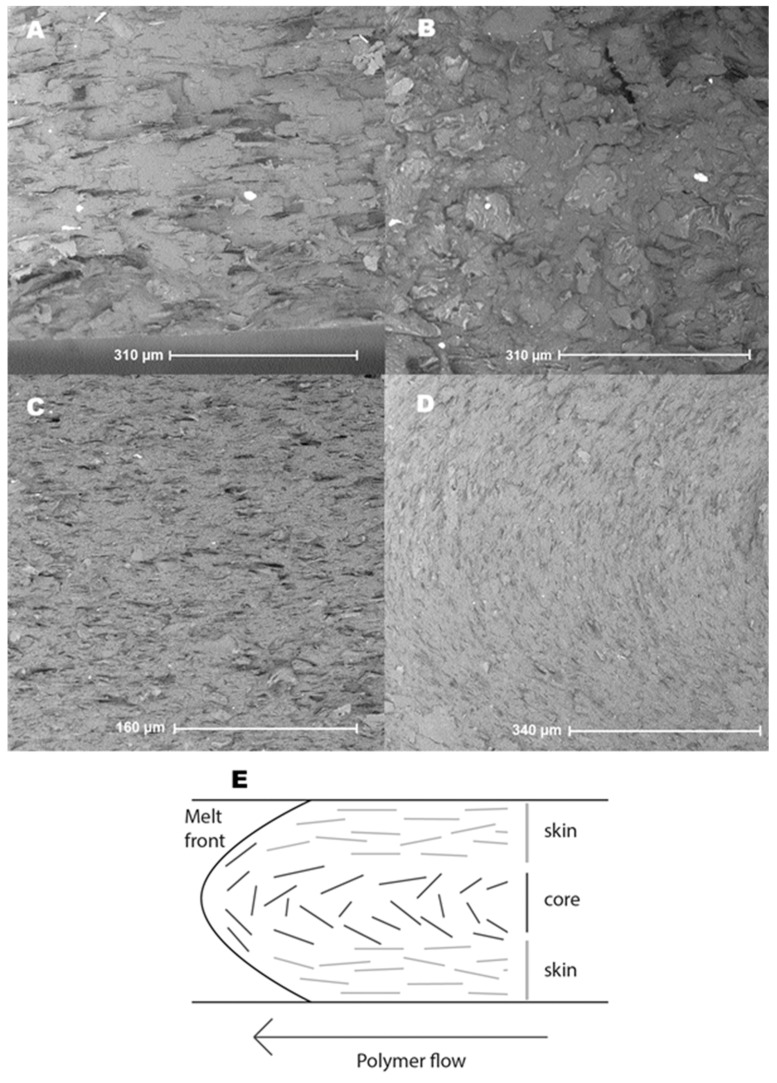
SEM image of ABS with 20 m% of graphite; (**A**) near the edge, where the skin effect is clearly visible and (**B**) at the center of the sample, where more random orientation is visible; SEM image of PA6 with 20 m% of graphite (**C**) near the edge of the sample, where the same orientation as the ABS sample is visible and (**D**) at the center of the sample, where the fillers show a clearer orientation following the melt front; (**E**) global SEM interpretation: orientation of fillers due to the skin-core effect (exaggerated); the 2D graphite platelets are represented as 2D lines for simplification.

Figure 4 shows the corresponding SEM images (20 m%), where the injection direction is from right to left. Images A to C display composites with well-dispersed fillers. No clear coagulations of graphite are visible, as these could cause strong changes in the local thermal conductivity. Figure 4A was taken for ABS near the edge of the sample, hence, in the skin layer. Fillers clearly show strong alignment with the flow direction, improving in-plane and decreasing through-plane TC. This image also shows the 2D (platelet) character of the graphite filler. Figure 4B was taken at the center of the ABS sample or thus the core layer. The graphite fillers show no clear alignment or preferred direction and seem to be orientated randomly. This random orientation could be able to form a 3D interconnected network at the core, improving both in-plane as through-plane TC. Closer examinations of multiple SEM images, however, showed a distinguished pattern where the fillers in the core still oriented with the flow front but much less outspoken than the skin-layer orientation. Figure 4C,D shows the skin and core layer with PA6, with specifically the core layer of PA6 clearly showing the orientation with the melt front (Figure 4D). Much like Figure 4A, the skin effect is well visible in Figure 4C. An exaggerated representation of filler orientation after injection molding is shown in Figure 4E. It can be concluded that the effect of skin-core layer formation should not be underestimated for TC. For improved through-plane TC, the skin layer should be as small as possible. Alternatively, removal of the skin layers of less complex shapes could make it possible to achieve high through-plane TC, even at low filler amounts. To further support this claim, the top 1.5 mm was grinded off the samples. However, the through-plane TC or thickness of the sample were respectively too high or too low in order to be measured, as the probing depth exceeded the sample thickness.

### 3.6. Relevance of Morphology: Filler Size and Shape

Morphology can also be influenced by filler size. In order to eliminate the aforementioned (small) effect of crystallinity and other factors, amorphous PS 165N was chosen as matrix material and combined with graphite filler of two different sizes, henceforth called “macro-graphite” (used before; Table 3, Table 4, Table 5, Table 6 and Table 7) and “nano-graphite”. The different compositions and the TC of these compounds are given in Table 8, again selecting the optimal amount of 20 m% but also including additional data for 10 m% of filler. Upon comparing the macro-graphite at 20 m% with the nano-graphite at 20 m%, it can be noticed that the former performs better both in- and through-plane. This is more than likely due to the increased number of interfaces in the nano-composite compared with the macro-composite. Only the macromaterial can likely form more conductive paths. Upon combining nano with macro-graphite (overall still 20 m%), a decrease in both in-plane and through-plane TC can be noticed in Table 8. Increasing the amount of nano-graphite while decreasing the amount of macro-graphite causes the in-plane TC to fall further, since the existing percolation network in this direction becomes broken by removing macro-fillers, further highlighting the preference of macro-graphite.

**Figure 5:**
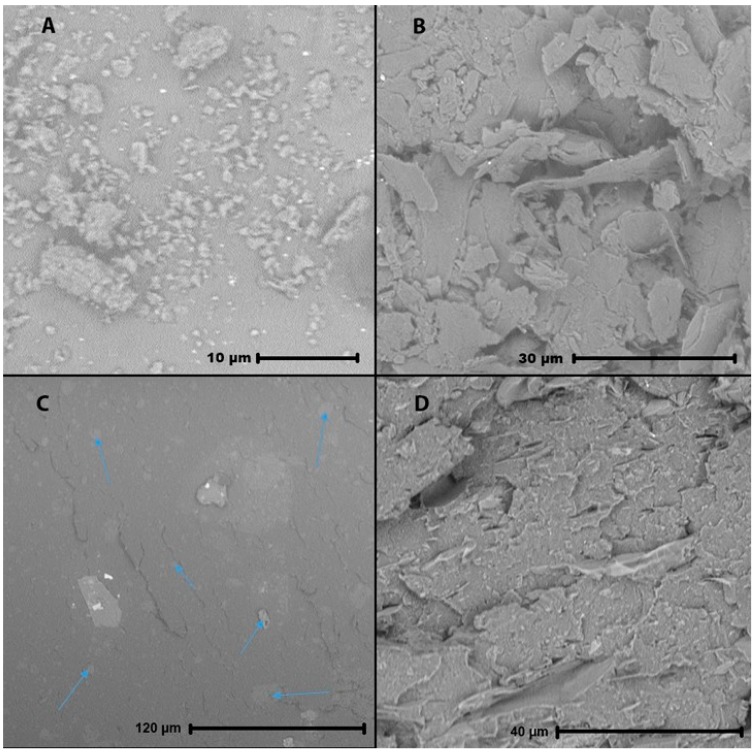
SEM images of (**A**) the nano-and (**B**) the macro-graphite, with spherical lumps vs. 2D-platelets; (**C**) shows 20 m% of nanofiller in PS 165N: particles are visible as slightly lighter spots, some indicated with arrows. Nanofillers are harder to detect in (**D**) 10 m% of each filler in PS 165N.

It should be put forward that also the shape of the fillers plays a role. Figure 5A,B show SEM-images of both fillers. While it is clear that the nano-fillers (A) are easily a factor 10 smaller (besides some clusters) than the macro-fillers (B), the nano-fillers appear as more or less spherical lumps, while the macro-fillers appear as 2D-platelets. The lumps will show less preferred orientation upon injection, besides having a more uniform TC individually. This can explain the smaller gap between in-plane and through-plane TC of the nano-composites compared with the macro-composites in Table 8. This could also explain why the nano-filler at 10 m% has a higher through-plane TC than the macro-filler at the lower amount of 10 m%. Figure 5C,D show SEM images for nano-fillers (20 m%) and a combination of nano and macro-fillers (10/10 m%) in a PS matrix. The nano-fillers in (C) still appear as more or less spherical clusters, some indicated with arrows, whereas the macro-fillers in (D) appear as two-dimensional platelets. The nano-fillers are not noticeable in the rough sample fracture where macro-fillers are present as well. Hence, the shape of the macro-fillers is more suited for a TC increase, provided that the correct filler amount (20 m%) is selected.

## 4. Conclusions

The effect of average molar mass, crystallinity, moisture, matrix type, and filler amount and type on the (overall) TC was studied based on commercial polymers and their composites that were produced on a semi-industrial scale, using twin-screw compounding and injection rather than lab-scale techniques such as solvent mixing and compression molding. The effect of average molar mass was found to be insignificant, whereas a higher crystallinity does imply a higher TC although it is not the only contributor. Further increase of the crystallinity for instance by annealing is more relevant in view of the increase of mechanical properties. Notably, moisture has no true effect, which highlights the potential of polymer composites for thermal management applications.

A general trend of higher in-plane and lower through-plane TC was noticed in all polymers, caused by the flow during the processing. The consideration of graphite fillers (20 m%) can increase both the in- and through plane TC. Although HDPE and PA6 composites generally showed a higher TC than other polymers with a better overall performance than for instance previously developed PP-carbon fiber-carbon nanotube composites, it is non-trivial to notice emerging trends in the TC of all the composites. This is likely because of the complexity of the whole system, where the skin-core effect also plays an important role. This skin-core effect has a substantial influence on the overall TC of the composite. An increase up to 100% in through-plane TC is noticed upon measuring deeper, hence, in the core-layer. For thermal applications requiring a high through-plane TC, the skin layer should therefore be as thin as possible. Removing the skin layer after injection could thus be considered for improved performance, at least if a basic shape is considered.

Macro-fillers generally perform the best, showing a higher in-plane and through-plane TC at 20 m%. Adding nano-fillers decreases both TCs, likely because they have more interfaces to cross, resulting in more phonon scattering. SEM images revealed that, besides the size, macro- and nano-fillers show a different shape. The spherical shape of the nano-filler causes a lower increase in the in-plane TC, since spheres are less affected by flow and thus orientation. The low aspect-ratio also makes it more difficult to form thermal conductive networks in contrast to the preferred macro-fillers.

## Figures and Tables

**Table 1:** Melt enthalpy of 100% crystalline material [37,38].

**Polymer matrix**	HDPE	PP	PLA	PA6
**Enthalpy of fusion (J·g^−1^)**	293	207	94	230

**Table 2:** Thermal conductivity (TC) of PS with different average molar mass; isotropic (bulk) values; no fillers; no significant differences are recorded; TC at half the sample thickness.

Grade	TC (W·m^−1^K^−1^)
PS 124N	0.1768
PS 165N	0.1761

**Table 3:** Crystallinity (*x*_c_) and thermal conductivity (TC) of several commercial polymers without nucleation agent and filler; polymers ranked according to increasing TC; annealing for second PLA; a higher crystallinity helps to improve the TC but is a such only a contributing factor; TC at half the sample thickness.

Polymer	*x*_c_ (%)	TC (W·m^−1^K^−1^)
PS 165N	0	0.1768
ABS	0	0.1891
PLA (amorphous)	0	0.2082
PLA (semi-crystalline)	48	0.2202
PP	44	0.2456
PA6	16	0.3271
HDPE	62	0.4657

**Table 4:** Crystallinity (*x*_c_; %) and thermal conductivity (TC; W·m^−1^K^−1^) of PA6, annealed PA6 (PA6-a), PLA and annealed PLA (PLA-a) with different amounts of nucleating agents (N.A.); 0.2% N.A. is sufficient; to increase *x*_c_; *x*_c_ increases are mainly useful for controlling mechanical properties as similar TC values are obtained; no filler; TC at half the sample thickness.

		0% N.A.	0.2% N.A.	0.5% N.A.	1.0% N.A.
**PA6**	TC	0.3271	0.3154	0.3183	0.3210
	*x*_c_	16	24	25	27
**PA6-a**	TC	0.3352	0.3273	0.3239	0.3355
	*x*_c_	31	31	29	31
**PLA**	TC	0.2082	0.2092	0.2124	0.2138
	*x*_c_	0	18	17	19
**PLA-a**	TC	0.2202	0.2246	0.2246	0.2240
	*x*_c_	48	50	49	49

**Table 5:** Thermal conductivity (TC) of wet, dry and conditioned PA6 samples (no filler). Very small changes are observed, which is interesting for thermal management systems; TC at half the sample thickness.

PA6	Conditioned	Dry	Wet
**TC (W·m^−1^K^−1^)**	0.3273	0.3196	0.3142

**Table 6:** Thermal conductivity (TC) of virgin polymers and their composites materials, with different amounts of fillers alongside relative increases (with respect to virgin polymer); no nucleation agent or annealing; virgin materials were measured as isotropic, hence, the same in-plane and through-plane values: TC at half the sample thickness.

	**In-Plane TC (W·m^−1^K^−1^)**			**In-Plane TC (Relative in %)**		
**m% of filler**	0	10	20	0	10	20
**HDPE**	0.4657	1.5996	4.3084	0	243	825
**ABS**	0.1891	0.9485	3.0778	0	401	1527
**PP**	0.2456	1.0998	2.1126	0	347	760
**PA6**	0.3271	1.4975	4.3415	0	35	1227
**PS (165N)**	0.1761	0.9342	2.9346	0	43	1566
**PLA**	0.2082	1.2724	4.3328	0	51	981
	**Through-Plane TC (W·m^−1^K^−1^)**			**Through-Plane TC (Relative in %)**		
**m% of filler**	0	10	20	0	10	20
**HDPE**	0.4657	0.5499	0.6957	0	18	49
**ABS**	0.1891	0.2129	0.2526	0	12	33
**PP**	0.2456	0.176	0.1902	0	−28	−22
**PA6**	0.3271	0.4444	0.4711	0	35	44
**PS (165N)**	0.177	0.1676	0.2576	0	−5	45
**PLA**	0.2082	0.2007	0.2464	0	−3	18

**Table 7:** Thermal conductivities (TCs) as a function of probing depth to identify the relevance of gradients (cf. Figure 3); in Table 2, Table 3, Table 4, Table 5 and Table 6, focus was on values with a minimization of the skin-core layer by a regulated probing depth at half the sample thickness; at lower probing depths, the influence of the skin layer becomes clear.

	Probing Depth (mm)	Through-Plane TC	In-Plane TC
**PLA 20 m%**	1.21	0.2081	4.5699
	1.94	0.2385	4.4190
	2.99	0.4251	3.1111
**ABS 20 m%**	1.10	0.1626	3.9392
	2.04	0.2487	3.0215
	3.51	0.5131	1.7326

**Table 8:** Thermal conductivities (TCs; W·m^−1^K^−1^) of PS 165N composites with different amounts of macro- and nano-graphite; the use of macro-graphites (Table 3, Table 4, Table 5, Table 6 and Table 7) is preferred (20 m%); TC at half the sample thickness.

**nano/macro (m%/m%)**	0/10	0/20	5/15	10/10	15/5	20/0	10/0
**In-plane TC**	0.9341	2.9345	1.4434	1.2000	0.9236	0.4526	0.4409
**Through-plane TC**	0.1676	0.2576	0.1809	0.1984	0.2120	0.228	0.1905

## Supplementary Materials

The following are available online at http://www.mdpi.com/2073-4360/11/1/87/s1, Figures S1 and S2: additional DSC results for study effect crystallinity.
