# Supplementary material for: Effect of Matrix and Graphite Filler on Thermal Conductivity of Industrially Feasible Injection Molded Thermoplastic Composites
Source: Polymers (Basel). 2019 Jan 8;11(1):87. doi: 10.3390/polym11010087 (PMC6402235; doi:10.3390/polym11010087)
Supplement: Supplementary file 1 [file polymers-11-00087-s001.pdf]

# Supporting Information for 'Effect of matrix and graphite filler on thermal conductivity of industrially feasible injection molded thermoplastic composites'

**Tom Wieme [1], Lingyan Duan [1], Nicolas Mys [1], Ludwig Cardon [1*] and Dagmar R. D'hooge[2,3,*]**

[1] Centre for Polymer and Material Technologies, Department of Materials, Textiles and Chemical Engineering, Ghent University, Technologiepark 915, B-9052 Zwijnaarde (Ghent)
[2] Laboratory for Chemical Technology, Department of Materials, Textiles and Chemical Engineering, Ghent University, Technologiepark 914, B-9052 Zwijnaarde (Ghent), Belgium
[3] Centre for Textile Science and Engineering, Department of Materials, Textiles and Chemical Engineering, Ghent University, Technologiepark 907, B-9052 Zwijnaarde (Ghent), Belgium
* Correspondence: dagmar.dhooge@ugent.be ; ludwig.cardon@ugent.be

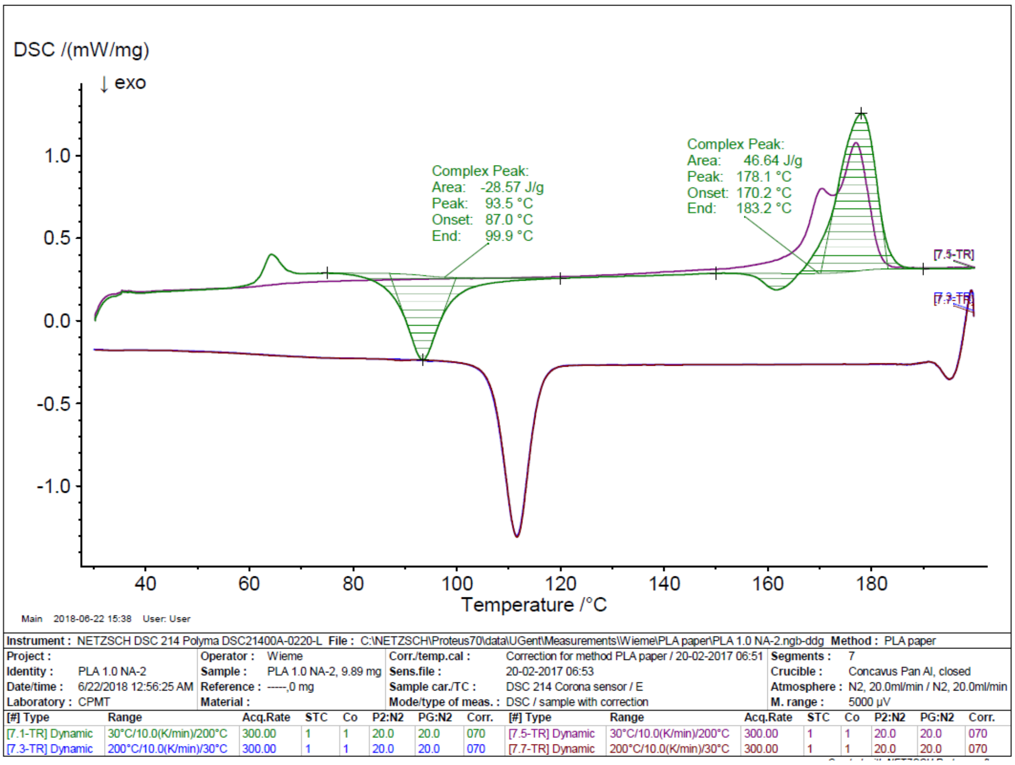


**Figure S1: DSC diagram of PLA with 1 m% nucleating agent before annealing. The first run (green) clearly shows cold crystallization**

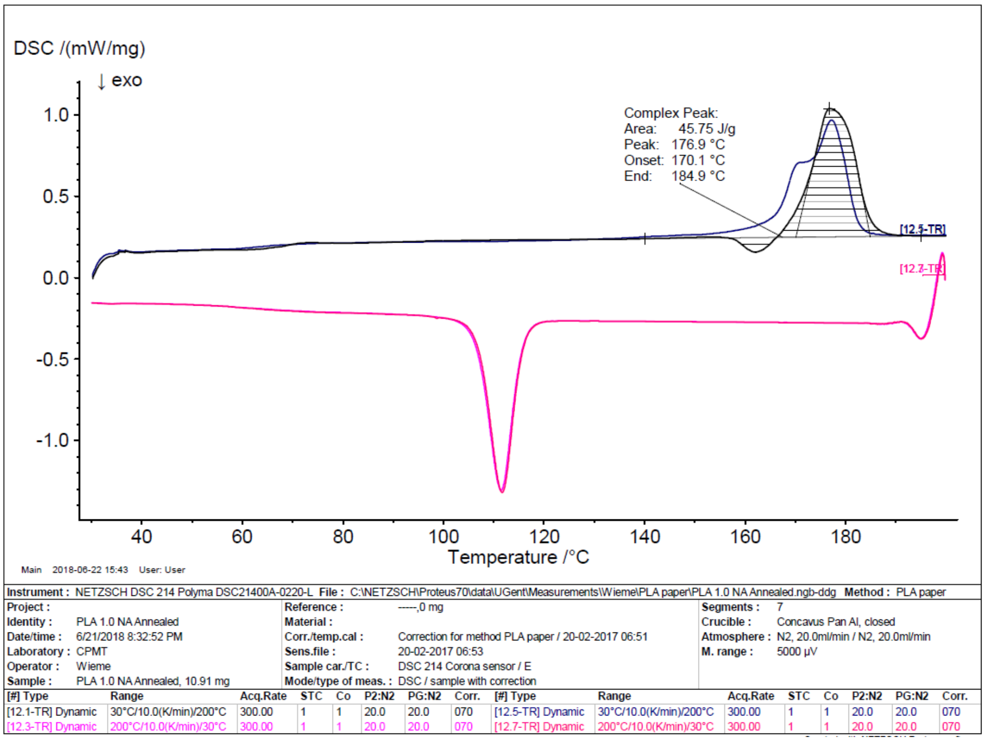


**Figure S2: DSC diagram of PLA with 1 m% nucleating agent after annealing. The first run (black) shows no cold crystallization.**
